# *Bacillus sphaericus* Binary Toxin Elicits Host Cell Autophagy as a Response to Intoxication

**DOI:** 10.1371/journal.pone.0014682

**Published:** 2011-02-14

**Authors:** Onya Opota, Nils C. Gauthier, Anne Doye, Colin Berry, Pierre Gounon, Emmanuel Lemichez, David Pauron

**Affiliations:** 1 Institut National de la Recherche Agronomique, UMR Interactions Biotiques et Santé Végétale, INRA 1301-CNRS 6243-Université de Nice Sophia Antipolis, Sophia Antipolis, France; 2 INSERM, U895, UNSA, Centre Méditerranéen de Médecine Moléculaire, C3M, Toxines Microbiennes dans la relation hôte pathogènes, Nice, France; 3 Cardiff School of Biosciences, Cardiff University, Cardiff, United Kingdom; 4 Centre Commun de Microscopie Electronique Appliquée, Faculté des Sciences, Université de Nice Sophia Antipolis, Nice, France; Iowa State University, United States of America

## Abstract

*Bacillus sphaericus* strains that produce the binary toxin (Bin) are highly toxic to *Culex* and *Anopheles mosquitoes*, and have been used since the late 1980s as a biopesticide for the control of these vectors of infectious disease agents. The Bin toxin produced by these strains targets mosquito larval midgut epithelial cells where it binds to Cpm1 (*Culex pipiens* maltase 1) a digestive enzyme, and causes severe intracellular damage, including a dramatic cytoplasmic vacuolation. The intoxication of mammalian epithelial MDCK cells engineered to express Cpm1 mimics the cytopathologies observed in mosquito enterocytes following Bin ingestion: pore formation and vacuolation. In this study we demonstrate that Bin-induced vacuolisation is a transient phenomenon that affects autolysosomes. In addition, we show that this vacuolisation is associated with induction of autophagy in intoxicated cells. Furthermore, we report that after internalization, Bin reaches the recycling endosomes but is not localized either within the vacuolating autolysosomes or within any other degradative compartment. Our observations reveal that Bin elicits autophagy as the cell's response to intoxication while protecting itself from degradation through trafficking towards the recycling pathways.

## Introduction

*Bacillus sphaericus* is one of the few biopesticides available for controlling *Culex* and *Anopheles* mosquitoes, which are vectors of human diseases such as West Nile fever and malaria [1], [2]. Bacillus sphaericus can produce different types of insecticidal proteins: the mosquitocidal toxins (Mtx) produced during vegetative growth and the binary toxin (Bin) produced during sporulation. Bin-producing strains are by far the most toxic and kill mosquito larvae within twenty-four to forty-eight hours [3]. Bin is synthesized in a parasporal crystalline inclusion as two protoxins, pro-BinA and pro-BinB. Upon ingestion by mosquito larvae, the crystal is solubilized by the alkaline pH of the digestive fluid and the protoxins are subsequently processed by proteolytic cleavage, leading to activated BinA and BinB that target the midgut epithelial cells. A single Bin-binding receptor protein has been identified in the brush-border membrane of epithelial cells of mosquito larvae: Cpm1 (*Culex pipiens* maltase 1) in *Culex* mosquitoes [4], [5] and its orthologue Agm3 (*Anopheles gambiae* maltase 3) in *Anopheles gambiae* [6]. Cpm1 and Agm3 are digestive enzymes anchored to the plasma membrane by a glycosylphosphatidylinositol anchor (GPI). In *Culex* species the binding to Cpm1 is triggered by BinB [4], [7], [8]; then BinA docks to the receptor-bound BinB and triggers toxicity, as reported for several A-B toxins [9]. The emergence of Bin resistant mosquito populations, which threatens the usefulness of this biopesticide, increases the necessity to fully understand its mode of action [3].

Electrophysiological analyses performed on cultured *Culex quinquefasciatus* cells and on large unilamellar phospholipids vesicles (LUVs) have shown the ability of Bin to induce channel formation [10], [11]. By expressing Cpm1 in the mammalian epithelial cell line MDCK (Maldin and Darby canine kidney) we have recently demonstrated the contribution of the Bin receptor Cpm1 to the formation of pores. While Bin had no effect on untransfected MDCK cells, it induced the formation of cationic channels in MDCK-Cpm1 [12]. When expressed in MDCK cells, Cpm1 fully retained its biochemical and functional characteristics such as GPI-anchoring to the apical side of polarized cells, enzymatic activity and high binding affinity to Bin. Moreover, we showed that Cpm1 is concentrated in lipid raft microdomains which may facilitate the oligomerization of the toxin/receptor complex and contribute to the pore formation process [12].

The synthesis of pore forming toxins is a strategy widely used by pathogenic bacteria to trigger their virulence [13]. Nevertheless, pore-forming toxins display numerous modes of action ranging from the formation of lytic pores in the plasma membrane to the translocation of components displaying enzymatic activities or able to interfere with intracellular signalling pathways [9], [14]. It has previously been shown that the lethal effect of Bin is not associated with epithelial cell lysis or epithelium disruption and, to date, the mechanism by which Bin kills mosquito larvae remains unsolved [3], [15]. The midgut epithelial cells of mosquito larvae intoxicated with Bin display several cytopathologies affecting the microvilli, the mitochondria and the rough endoplasmic reticulum but the most dramatic feature of Bin intoxication is the appearance of abnormal, electron-clear vacuoles indicating an important cellular stress [15], [16], [17]. Remarkably, Bin-induced cytoplasmic vacuolation described in Culex mosquito midgut epithelial cells, was also found in Bin treated MDCK-Cpm1 cells [12]. In the present study, we take advantage of the MDCK-Cpm1 cell line to perform the first study investigating the Bin cellular mode of action. We demonstrate for the first time that Bin promotes the appearance of large vacuoles that present features of autolysosomes. Strikingly, we found that while this vacuolation is partially reversed over time, it continues to affect a significant subset of the cell population. A careful time-lapse videomicroscopic analysis reveals that in fact, dividing cells were preferentially affected by the vacuolation in an unreported phenomenon that we have called post-mitotic vacuolation. Furthermore, we found that induction of autophagy was elicited by Bin in intoxicated cells and that stimulation of autophagy prior to intoxication inhibited autolysosome vacuolation. Our findings suggest that the vacuolation concerned the initiation of the autophagy process. Furthermore, the study of the intracellular trafficking of fluorescent Bin derivatives revealed that after endocytosis, the toxin reached recycling endosomes but was not routed toward autophagosomes, autolysosomes or lysosomes. In addition, Bin trafficking reveals how a bacterial toxin can conserve its toxic potential by avoiding the target cell degradative pathway it elicits.

## Results

### Bin induces the vacuolation of a compartment that possesses autolysosomal characteristics

**Figure 1:**
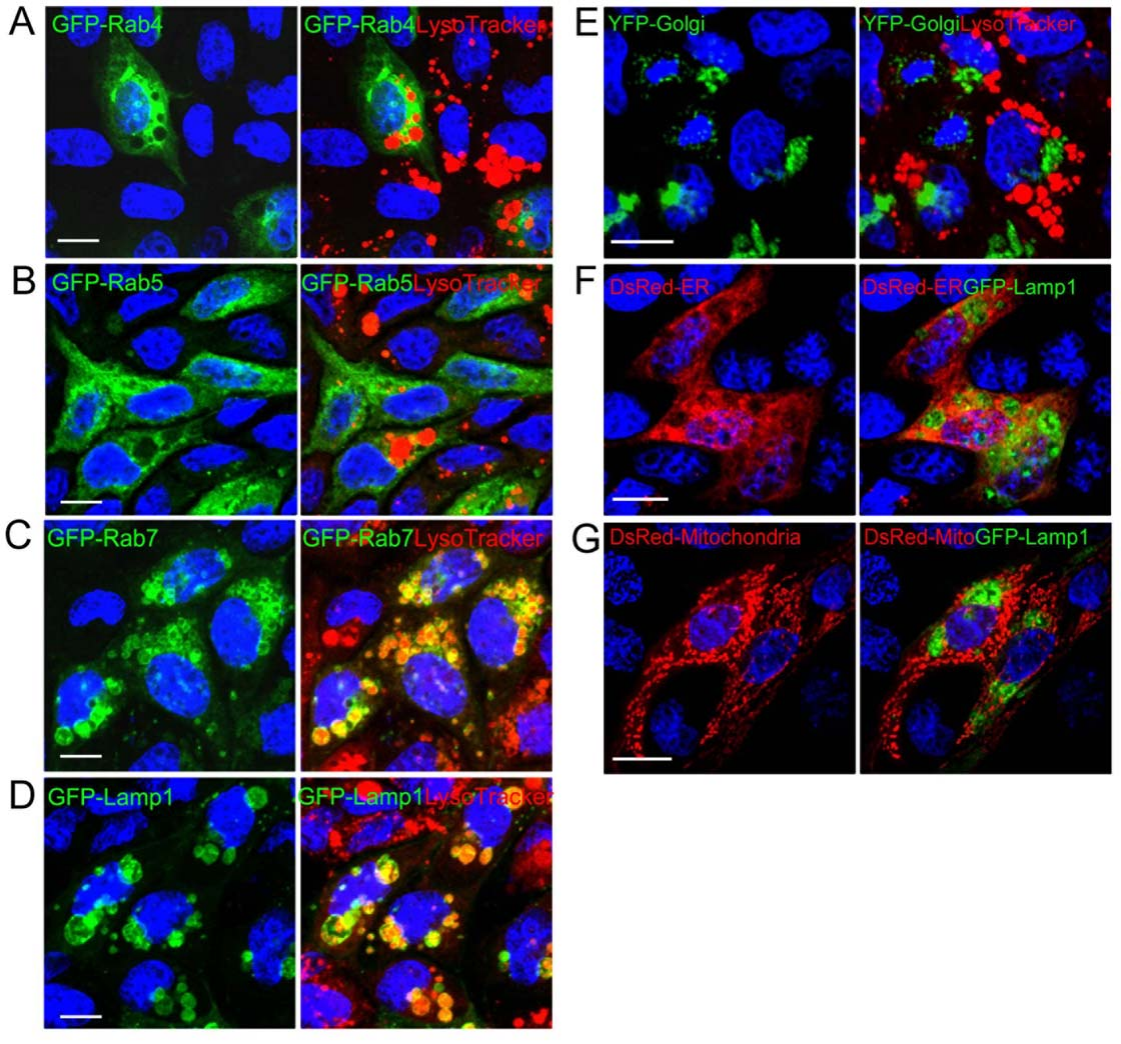
The giant vacuoles induced by Bin possess characteristics of late endocytotic and lysosomal compartments. (A-E) MDCK-Cpm1 cells were transfected with GFP-Rab4, GFP-Rab5, GFP-Rab7, GFP-Lamp1 or YFP-Golgi and treated with BinA and BinB for 6 h. LysoTracker (1 µM) was added to the medium 5 min before the end of the treatment. (F and G) MDCK-Cpm1 cells were co-transfected with DsRed2-Endoplasmic Reticulum or DsRed2-Mitochondria and GFP-Lamp1 before intoxication. Bin-induced vacuoles stained with LysoTracker or GFP-Lamp1 do not colocalize with marker of Golgi apparatus, ER and mitochondria. Bars, 10 µm.

To determine the origin of the vacuolating structures induced by Bin in MDCK-Cpm1 cells we expressed organelle-specific markers fused with fluorescent proteins. While Bin-induced vacuoles could be distinguished with the acidotrophic marker LysoTracker, we found that the early endosome marker Rab5 and the recycling endosome marker Rab4 were excluded from these vacuoles (Figs. 1 A and B, Fig. S3 and video S6). In contrast, the late endosome marker Rab7 and the lysosome marker Lamp1 (Lysosome-associated membrane protein 1) were clearly associated with the membrane of the LysoTracker-positive vacuoles (Figs. 1 C and D, Fig. S3 and video S5). Markers of the Golgi apparatus, the endoplasmic reticulum and the mitochondria were not found associated with the giant vacuoles decorated with Lamp1 and stained with LysoTracker (Figs. 1 E to 1G). We also examined the distribution of the cytoskeleton network in vacuolated cells. Treating cells with Bin did not affect the microtubule architecture or the actin stress fiber network. Nevertheless, when examining cells under various focal planes we found actin filaments surrounding the membrane of Bin-induced vacuoles (Fig. S1).

**Figure 2:**
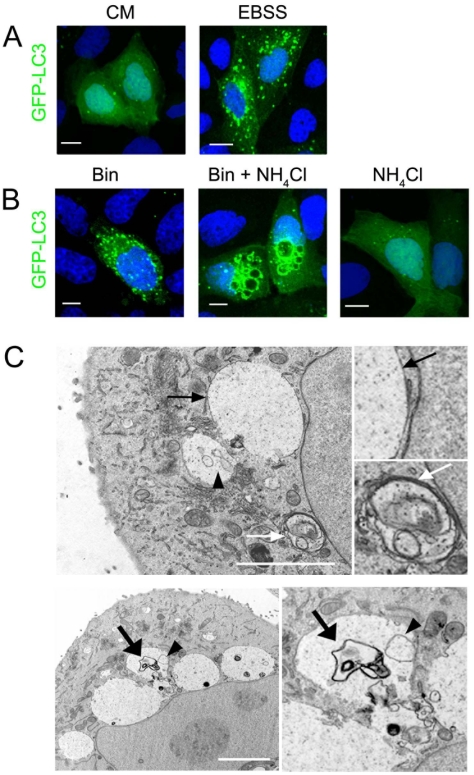
Bin-induced vacuoles originate from autolysosomes. (A) MDCK-Cpm1 cells transfected with GFP-LC3 and grown in complete medium (CM) or in nutrient-free medium (EBSS). Diffuse GFP-LC3 pattern seen in CM-grown cells is representative of non-autophagic cells. In contrast, GFP-LC3 punctated pattern seen in EBSS-grown cells is characteristic of autophagic cells. (B) Localization of the autophagic marker GFP-LC3 in cells treated with Bin for 6 h. To allow the detection of GFP-LC3 associated with autolysosomes NH_4_Cl (5 mM, final concentration) was added to the culture medium during the last hour of intoxication. A similar treatment with NH_4_Cl in the absence of toxin had no toxic effect on the cells and did not induce any change in GFP-LC3 pattern. (C) Bin-treated cells were processed for EM analysis as described in Experimental procedures. Vacuoles induced by Bin are electron-clear matrices enclosed by a single membrane (black arrows). In contrast, autophagosomes are double membrane vesicles (white arrows). Bin-induced vacuoles often contained vesicle-like structure (arrow-head) and partially degraded material (large black arrows). Bars, 5 µm (A and B); 2 µm (C).

Because of the convergence between the endocytic and the autophagic pathways [18], [19], we investigated the possibility that Bin-induced vacuoles displayed both autophagic and late endocytic/lysosomal features. The microtubule-associated protein light chain 3 (LC3), is a mammalian homologue of Atg8 which is a constituent of the autophagic vesicles in yeast [20], [21]. When autophagy is induced, LC3 is processed from a cytosolic form, LC3-I, to the LC3-II form that is associated with the autophagic vesicle membranes: elongating membranes and autophagosomes. In autophagic cells expressing GFP-LC3, these structures can be detected by fluorescence microscopy as dots (also named GFP-LC3 positive cells), while in non-autophagic cells GFP-LC3 presents a diffuse cytosolic pattern [20], [22] (Fig. 2 A). While we could find Bin treated cells presenting an autophagic pattern, we could not detect any GFP-LC3 signal that could be related to an association with the membrane of the giant vacuoles (Fig. 2B).

In order to initiate the autophagic catabolic step, autophagosomes fuse with lysosomes to generate autolysosomes where degradation ultimately occurs. Notably most of the GFP-LC3 is degraded, and its signal is lost. Adding NH_4_Cl to the medium reduces the luminal acidity of the vesicles so that the degradation of the GFP-LC3 marker is prevented and the detection of autolysosomes is increased [23], [24], [25]. A similar treatment allowed us to detect a GFP-LC3 signal associated with the membrane of Bin-induced vacuoles and inside these vacuoles (Fig. 2B). As a control, cells treated with NH_4_Cl alone did not display any vacuolation (Fig. 2B). At the ultrastructural level, Bin-induced giant vacuoles looked like homogeneous electron-clear matrices limited by a single membrane characteristic of autolysosomes, in contrast to autophagosomes, which are enclosed by a double membrane (Fig. 2C). In addition, these vacuoles often contained partially degraded material and vesicular like structures that can be related to autophagic bodies [26] (Fig. 2C). Finally, neither homogeneous cytosol containing a low number of organelles nor nuclear fragmentation, hallmarks of apoptosis or necrosis could be observed. Taken together these results support the hypothesis that Bin-induced vacuoles originate from an autolysosomal compartment.

### Bin-induced vacuoles form transiently

**Figure 3:**
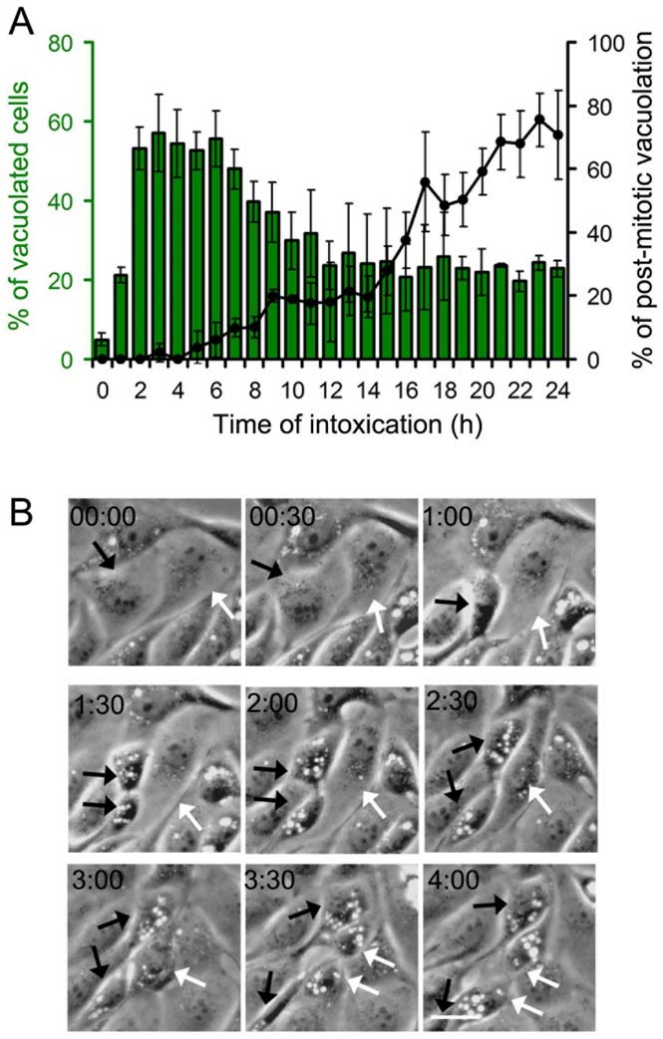
Dynamics of Bin-induced vacuolation. (A) Bin-treated (for 24 h) subconfluent MDCK-Cpm1 cells were submitted to time-lapse phase contrast video microscopy, as exemplified in video S1. At the indicated period of time the percentage of MDCK-Cpm1 cells displaying at least one vacuole was determined. Mean values ± SD, n  =  3. In the same videos, the percentage of cells that present post-mitotic vacuolation among the cells that display at least one vacuole, as illustrated in (B) was determined. Mean values ± SD, n  =  3. (B) Bin-treated cells displaying the post-mitotic vacuolation phenomenon; images selected from video S2. The selection starts 15h after intoxication, intervals in hours. Black and white arrows pinpoint two dividing cells. Bars, 10 µm.

In order to determine the dynamics of Bin-induced vacuolation of MDCK-Cpm1 cells we proceeded to time-lapse video microscopy. Vacuoles appeared within the first hour of intoxication in a subset of the cell population and rapidly increased in a time-dependent manner, finally affecting more than 50% of the cell population (Fig. 3A and Video S1). Strikingly, this vacuolation was transient, as the number of vacuolated cells started to decrease after 6 h of intoxication. Interestingly, at late time points of intoxication the vacuolation still affected about 25% of the cell population (Fig. 3A). By looking at individual cells we found that vacuolation could be reversed within a few hours. However, as shown in video S1 and S2 and figure 3B, we observed that the vacuolation reappeared in dividing cells and was dramatic after cytokinesis. We named this newly described phenomenon post-mitotic vacuolation. In fact, the number of cells vacuolating after mitosis increased as a function of time, and 24 h after intoxication almost all of the vacuoles were detected in post mitotic cells (Fig. 3 A). These results suggest that Bin-induced vacuolation is a transitory phenomenon that is recurrent in newly-divided cells.

### Induction of autophagy contributes to reversion of Bin-induced vacuolation

**Figure 4:**
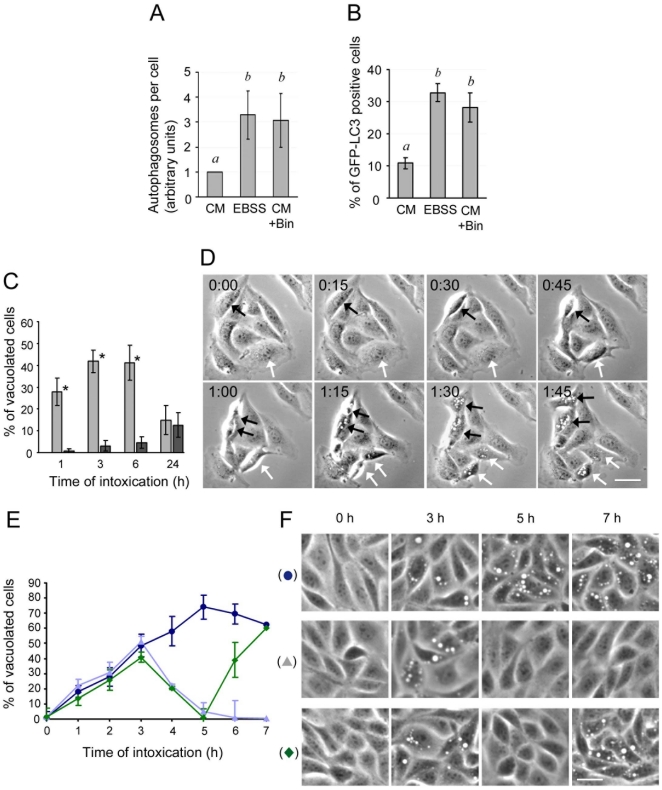
Autophagy is enhanced in Bin treated cells and contributes to vacuolation reversion. (A) MDCK-Cpm1 cells were grown for 6 h in complete medium in the absence (CM) or the presence of Bin (CM + Bin), or in nutrient-free medium in the absence of the toxin (EBSS), and then processed for EM analysis. Pictures from randomly chosen EM sections were analyzed for the presence of autophagosomes as defined in figure 2. The results are expressed as the number of autophagosomes per cell. Mean values ± SD, n  =  3. Different letters indicate statistically significant differences; P<0.05. (B) MDCK-Cpm1 cells expressing GFP-LC3 were grown under the conditions described above. At the indicated time, the number of GFP-LC3 positive cells was scored. Mean values ± SD, n  =  3. Different letters indicate statistically significant differences; P<0.05. (C) Cells were grown in complete medium or in nutrient-free medium in the presence of toxin. At the indicated period of time, the percentage of vacuolating cells was determined. Mean values ± SD, n  =  3. Asterisks indicate statistically significant differences; P<0.05. (D) Images selected from time lapse videomicroscopy of Bin-treated cells in nutrient-free medium displaying the post-mitotic vacuolation phenomenon, the selection starts 20 h after intoxication (Video S3). Black and white arrows pinpoint two dividing cells that will display this phenomenon. Bars, 10 µm. (E) MDCK-Cpm1 cells were grown in complete medium for 3 h (Δ, ⧫, •). Then the toxin was washed out with either complete medium (•), or nutrient-free medium (Δ, ⧫). Two hours after washing, the cells were returned to CM (⧫). Mean values ± SD, n  =  3. (F) Representative pictures illustrating the various patterns of vacuolation corresponding to each medium condition.

In Bin-treated MDCK-Cpm1 cells we could detect many autophagosomes that were either isolated or initiating fusion with the enlarged autolysosomes induced by Bin, which raised the hypothesis of an autophagy induction. A quantitative analysis confirmed that the number of autophagosomes was significantly higher in Bin-treated cells than in untreated cells grown in complete medium (Fig. 4A). Autophagy can also be monitored by scoring the fraction of GFP-LC3 positive cells [20], [27]. Under starvation, the percentage of GFP-LC3 positive cells is dramatically enhanced as a result of the induction of autophagy (Fig. 4B). In Bin treated cells, the percentage of GFP-LC3 positive cells was similarly enhanced (Fig. 4B). The increase in both the number of autophagosomes and the number of GFP-LC3 positive cells strongly suggested that autophagy is induced in MDCK-Cpm1 cells exposed to Bin. We next probed the effect of the induction of autophagy before Bin treatment. Figure 4C shows that when Bin was added to cells cultured in nutrient-free medium, vacuolation was inhibited. However, the percentage of vacuolating cells increased in a time-dependent manner and ultimately affected there again a subset of the cell population as in figure 4C. Videomicroscopy analysis indicated that this late vacuolation induced by Bin in nutrient-free medium (Fig. 4D and Video S3), was almost exclusively the post-mitotic phenomenon described in cells intoxicated in complete medium (Fig. 3B and Video S1). These results demonstrate that induction of autophagy prior to intoxication protects cells from vacuolation but does not inhibit the post-mitotic vacuolation. Because autophagy has been shown to be inhibited in dividing cells we hypothesised that this could explain the recurrence of post-mitotic vacuolation [28]. To challenge this hypothesis MDCK-Cpm1 cells were cultured in complete medium with Bin such that, after 3 h, approximately 50% of the cells were vacuolated (Figs. 4E and F). When the toxin was removed by washing with complete medium, the number of vacuolated cells continued to increase until it reached a maximum about 2 h after washing. Thereafter the vacuolation slowly decreased and was completely absent by 48 h after washing (unpublished data). In contrast, when the cells were washed with a nutrient-free medium, the vacuoles disappeared within 2 h (Figs. 4E and F). In order to confirm that this reversion was due to starvation alone, cells were grown again in toxin-free complete medium and the vacuolation rapidly reappeared (Figs. 4E and F). These results demonstrate that up-regulation of autophagy inhibited Bin-induced autolysosomal enlargement and in contrast, the consequence of a down-regulation of autophagy, similar to that which occurs in dividing cells, is the reappearance of the vacuolation.

### Bin is internalized, but is not associated with the giant autolysosomes

**Figure 5:**
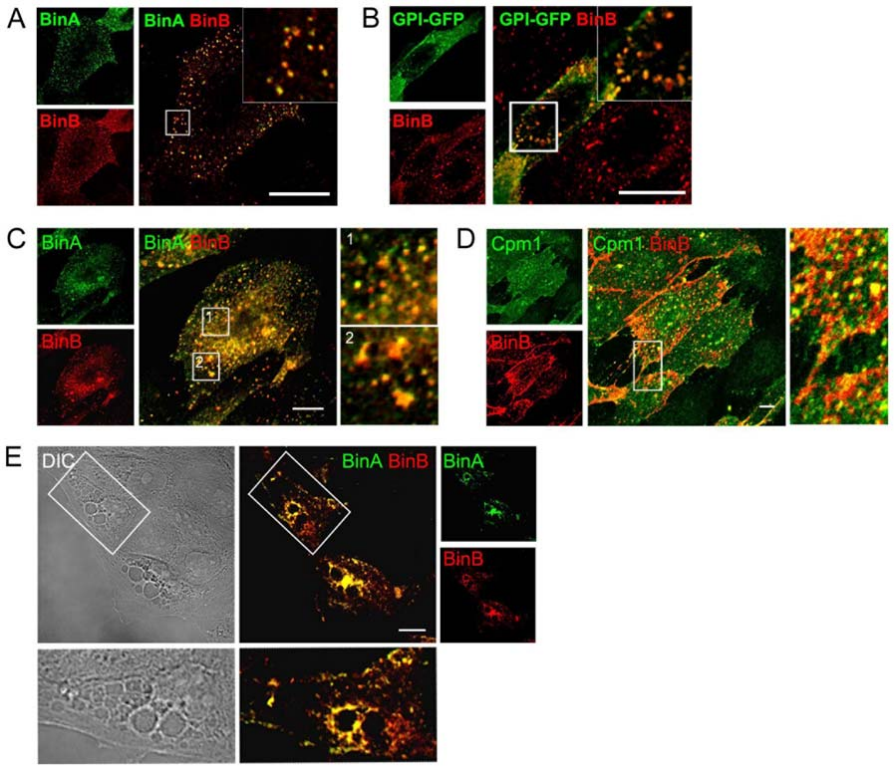
Internalized Bin is associated with its receptor but is not bound to vacuoles. (A, C and E) MDCK-Cpm1 cells were incubated with BinA labeled with Alexa 488 (BinA-Al^488^) and BinB labelled with Alexa 543 (BinB-Al^543^) at 37°C for 10 min (A), 30 min (C) or 6h (E) and processed for confocal microscopy analysis. At any time point, BinA-Al^488^ and BinB-Al^543^ were found colocalized in multiple intracellular vesicles. (B) Cells transfected with GPI-GFP and treated for 10 min with unlabelled BinA and BinB-Al^543^. Vesicles positive for BinB-Al^543^ were also stained by GPI-GFP. (D) Immunodetection of Cpm1 in MDCK-Cpm1 cells treated for 30 min with unlabelled BinA and BinB-Al^543^. Cpm1 signal and BinB-Al^543^ labeled vesicles were found colocalized. (E) BinA-Al^488^ and BinB-Al^543^-containing vesicles were found clustered between the vacuoles but not decorating the membrane of these vacuoles nor inside. Bars, 5 µm.

We next looked for Bin internalization in MDCK-Cpm1 cells by directly labelling BinA with Alexa 488 (Al^488^) and BinB with Alexa 543 (Al^543^). After 10 min of intoxication we could detect both BinA-Al^488^ and BinB-Al^543^ in small vesicles located mainly at the cell periphery (Fig. 5A). Then we proceeded to an intoxication with a combination of the BinB-Al^543^/BinA in cells transfected with a GPI-GFP construct. This construct consists of the decay accelerating factor (DAF) GPI anchor fused to GFP and serves as a valuable marker of compartments enriched in GPI-anchored proteins (GPI-APs) [29], [30]. At 10 minutes post-infection BinB-Al^543^ positive vesicles colocalized with the GPI-GFP marker suggesting that the toxin is internalized in association with its GPI-anchored receptor Cpm1 (Fig. 5B). After 30 min of intoxication, numerous endocytic vesicles containing both BinA-Al^488^ and BinB-Al^543^, as revealed by the colocalization of the two components, could be detected and were distributed throughout the cytoplasm (Fig. 5C). Immunodetection of the Bin receptor, the GPI-anchored protein Cpm1, revealed that it colocalized with the Bin endocytic vesicles (Fig. 5D). In vacuolated cells observed after 6 h of treatment, vesicles containing BinA-Al^488^ and BinB-Al^543^ were placed side by side with the vacuoles. Nevertheless the toxin was detected neither on the vacuole membranes nor inside the vacuoles (Fig. 5E, Fig.S4, video S7 and video S8). These results suggest that Bin is internalized in association with its receptor but is not targeted by the vacuolating autolysosomal compartment.

### Intracellular Bin is routed to the recycling pathway

**Figure 6:**
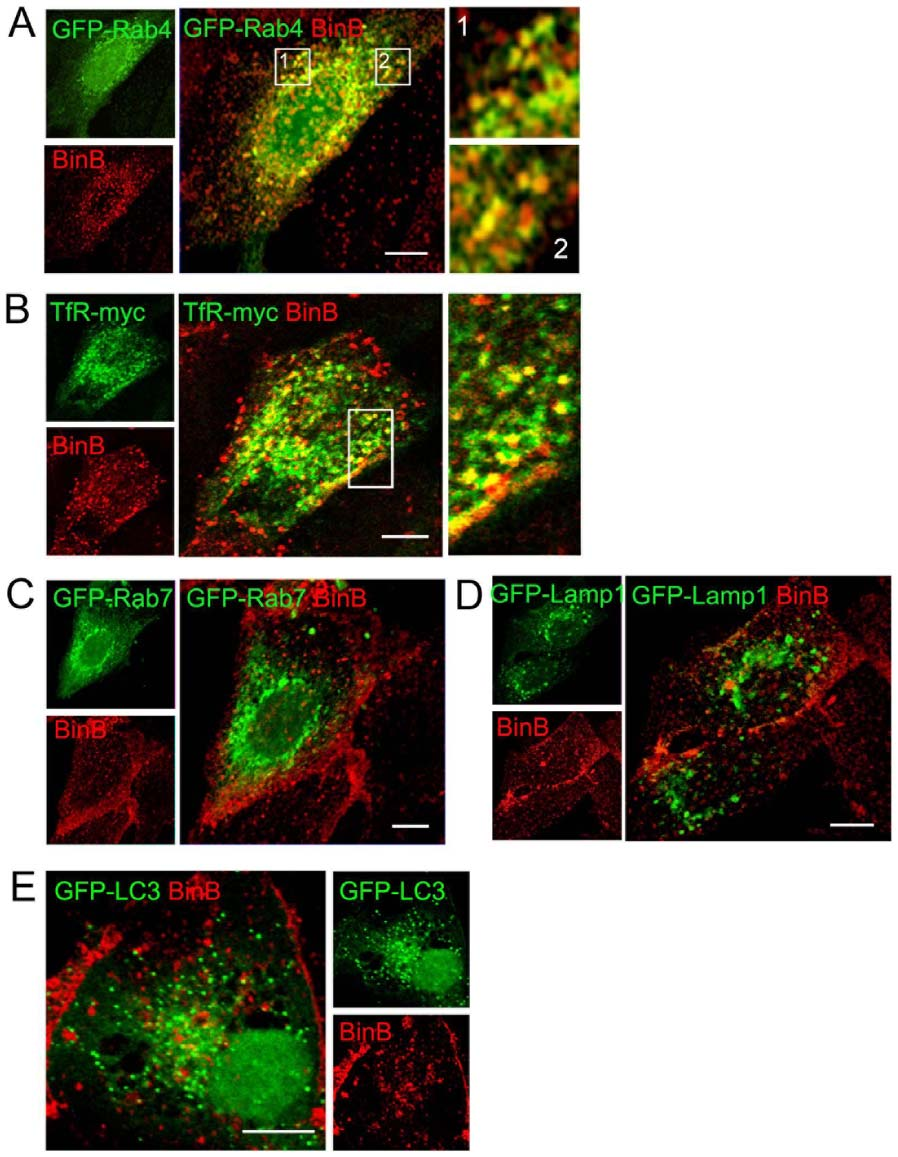
After internalization, Bin reaches recycling endosomes. MDCK-Cpm1 cells were transfected with markers of early and late endocytotic compartments, intoxicated with a mixture of unlabelled BinA and BinB-Al^543^ for 30 min and processed for confocal microscopy analysis. (A) BinB-Al^543^ fluorescent signal colocalized with GFP-Rab4, a typical marker of recycling compartments. (B) A significant colocalization was detected with TfR-myc that marks both endocytotic and recycling vesicles. (C - E) No colocalization was found with GFP-Rab7 and GFP-Lamp1 that marks late-endocytotic/lysosomal compartments and GFP-LC3 a marker of autophagic vesicles. Bars, 10 µm.

**Figure 7:**
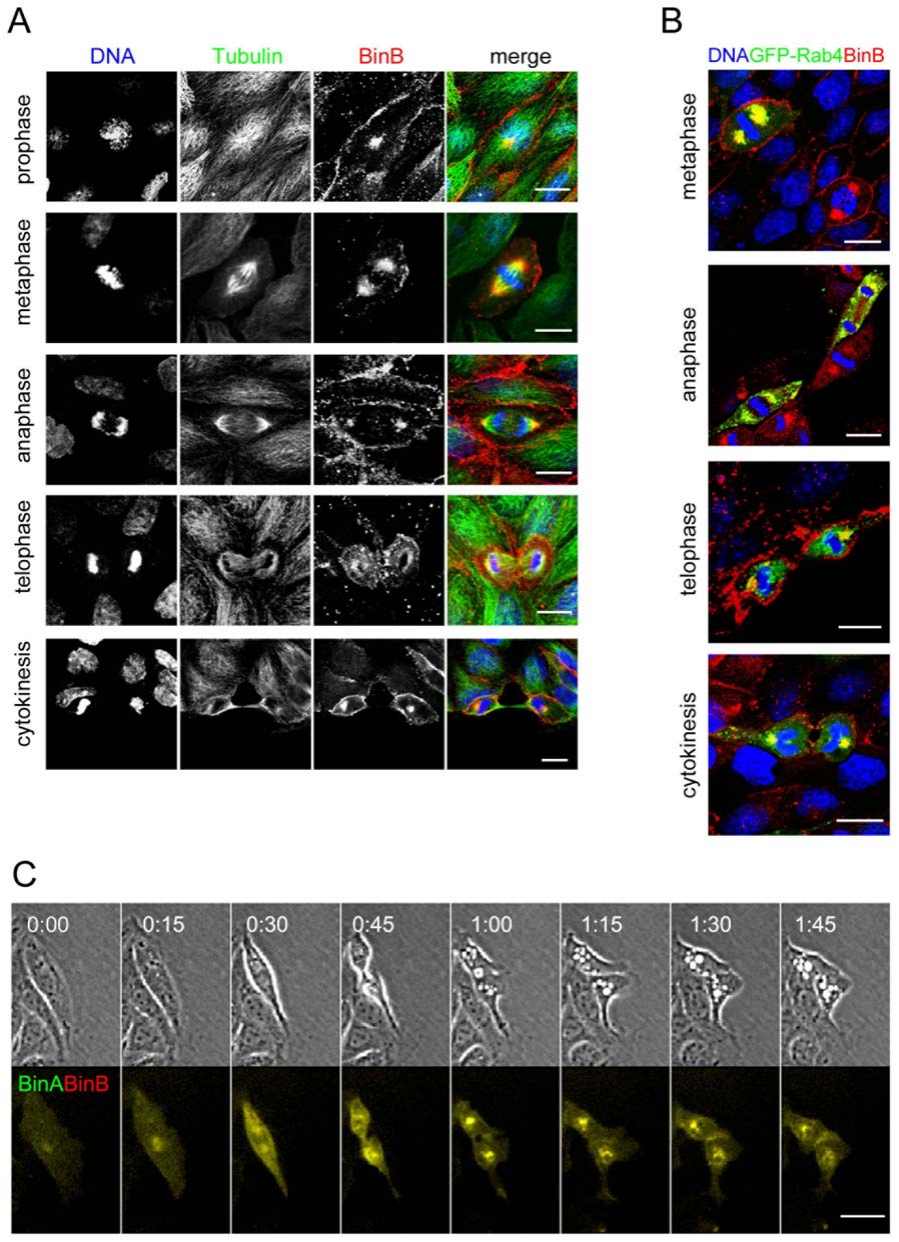
Fate of internalized toxin in dividing cells. (A) Immunodetection of β-tubulin and DNA staining were performed in MDCK-Cpm1 cells treated with unlabelled BinA and BinB-Al^543^ for 6 hours. (B) Cells were transfected with GFP-Rab4 then intoxicated as described above, fixed, and stained for DNA. Cells in the process of cell division were analyzed for the localization of the toxin. (C) Time lapse videomicroscopy analysis was performed on MDCK-Cpm1 cells treated with BinA-Al^488^ and BinB-Al^543^ for 24 h. The panel selected from video S4 shows a cell displaying post-mitotic vacuolation, the selection starts 16 hours after intoxication. While the cell was progressing through mitosis, internalized toxins were colocalized and partitioned in the daughter cells. Bars, 10 µm.

Because Bin was not associated with the vacuoles it induces, we tried to identify the intracellular compartment reached by the toxin. After 10 min of internalization, the vesicles stained with Bin were negative for the early endosomal markers GFP-Rab5 and Caveolin1-GFP, the transferrin receptor that marks both early and recycling endosomes, and GFP-Rab4 a marker of recycling endosomes (Fig. S2). Interestingly after 30 min of internalization, the vesicles containing Bin colocalized with the recycling endosomal marker GFP-Rab4 and the transferrin receptor, which exploits the recycling pathway during its trafficking (Figs. 6A and 6B). In contrast, no association could be observed with the late endosomal marker Rab7, the lysosomal marker Lamp1 or the marker of autophagic vesicles LC3 (Figs. 6C-E). We next investigated the fate of the toxin in dividing cells. The sequential phases of mitosis were identified via immunodetection of β-tubulin and DNA staining. During mitosis, the toxin localized to the microtubule organizing center (Fig. 7A). As shown in figure 7B, and consistent with figure 6A, recycling endosomes stained with GFP-Rab4 en route for partitioning into the daughter cells were located in the same area [31], [32]. Using time lapse microscopy, we confirmed that while cells progressed through mitosis, the internalized toxin was partitioned to the daughter cells and, when post-mitotic vacuolation appeared, the toxin was not associated at any time with the vacuoles but remained clustered in the free space (Fig. 7C; Video S4) as reported above (Fig. 5C). Taken together, these results established that the toxin is associated with recycling endosomes but not with the degradative compartments.

## Discussion

The molecular mechanism by which *B. sphaericus* binary toxin Bin kills mosquito larvae, particularly from the genera *Culex* and *Anopheles* is still undefined [3]. To date, the nature and the sequence of the cellular events leading to larval death remain unknown, cytoplasmic vacuolation being one of the major cytotoxic responses of *Culex* mosquitoes to Bin intoxication [15], [16]. In the present study, by using a system of MDCK cells expressing Bin receptor Cpm1 we show that Bin-induced giant vacuoles originate from autolysosomes, the final and degradative compartment of the autophagic pathway that result from the fusion of autophagosomes with lysosomes. This vacuolisation was not followed by cell death even at higher toxin concentrations. In Bin-treated cells, neither the distribution of the microtubule network nor the actin cytoskeleton that can induce the accumulation of very large autophagic vacuoles [33] was affected. Nevertheless, the detection of actin filaments surrounding the vacuolating autolysosomes suggests that the actin cytoskeleton may play a role in the invagination of the isolation membrane. Because the size of autolysosomes is maintained by the equilibrium between loading of autophagosomal content and the degradation process, one hypothesis was that Bin impaired this balance. Nevertheless, the material contained in the giant autolysosomes was partially degraded, indicating that the catabolic activity of autolysosomes remained effective. This does not exclude a delay between the generation of autolysosomes and the degradation process in this compartment that has been shown to generate abnormal vacuoles [25]. Interestingly, we have shown that Bin-induced vacuolation is a transient phenomenon, which after complete reversion in most of the cell remains recurrent in dividing cells in a previously undescribed phenomenon that we named post-mitotic vacuolation. Autophagy induction by starvation protected cells from vacuolation, but strikingly even under this condition the post-mitotic vacuolation was observed. During cell division and even in the absence of nutrient, autophagy is down-regulated with the aim of protecting chromosomes in the absence of the nuclear membrane [28]. Our observations suggest that the interruption of inhibition by autophagy that occurs when cells divide is responsible of the recurrence of the post-mitotic vacuolation.

Autophagy plays an efficient role in cellular defense against bacterial invasion [34], [35], [36]. Nevertheless, several invasive bacteria have developed strategies to escape autophagy degradation either through modification of autophagosome compartments, as with *Legionella pneumophila*, or by subverting autophagy, as with *Shigella flexneri* in MDCK cells [37], [38], [39]. Recently, the non-invasive bacteria *Vibrio cholerae*, *Helicobacter pylori* and *Bacillus anthracis* have been shown to modulate autophagy through the secretion of the pore forming toxins VCC, VacA and Anthrax respectively [40], [41], [42], [43]. An attractive hypothesis could be that the pore formation that can be detected by epithelial cells through the p38 mitogen-activated protein kinase (MAPK) activity [44], represents a signal danger that would elicit autophagy [13]. This hypothesis is supported by the requirement of VacA channel-formation activity to elicit autophagy in cells it intoxicates [41]. In agreement with this hypothesis, we found that the binding subunit BinB that is able by itself to disturb the membrane permeability [10], [11], was also capable of inducing an autolysosome enlargement (unpublished data). In contrast to the vacuolation induced by the mixture of the two components of Bin, vacuolation induced by BinB alone was rapidly and completely reversed, perhaps due to the instability of BinB derived pores.

Autophagy induced by VCC and by VacA have been shown to target and degrade the toxin and as a consequence, contribute to the intoxicated cell's survival [40], [41]. We found that Bin and its GPI-anchored receptor Cpm1 are rapidly internalized in an early endocytic compartment that did not present classical markers of early endocytic vesicles such as Rab5, caveolin-1 or the transferrin receptor. Nevertheless this compartment was enriched in GPI-APs. Such compartments from which GPI-APs can be directly targeted to recycling endosomes have been described by Sabharanjak *et* al. and are named GPI-APs-enriched early endosomal compartments, GEECs [45]. After endocytosis Bin would exploit this endocytic route to avoid degradation as it was associated with recycling endosomes rather than with the degradative compartments. This localization provides additional facilities to a bacterial toxin: Shiga toxin is hypothesized to traffic through recycling endosomes before reaching the Golgi apparatus with the aim of exploiting a retrograde transport in the secretory pathway [46]. Thus Bin could trigger its lethal effect beyond the epithelial monolayer by exploiting a transcytotic pathway from the apical membrane to the basolateral membrane that could include trafficking through recycling endosomes. Such a hypothesis is further prompted by the fact that cytopathologies affecting nervous cells and skeletal muscles have been reported following *Bacillus sphaericus* ingestion [47]. Recently it has been demonstrated that another *Bacillus sphaericus* toxin pair, Cry48/Cry49 that contains the Bin-like Cry49 protein, also elicits cytoplasmic vacuolisation, characteristic of Bin intoxication [48]. Thus, the mechanism of vacuole formation elucidated above may be exploited by other toxins from this organism.

In this study, we provide new insights into the strategies used by non-invasive bacteria to overcome cellular defense responses, i.e. a stimulation of autophagy and ability to subvert this degradation processes by trafficking in the recycling pathway. While the survival of invasive bacteria depends on their ability to counteract autophagic degradation [37], the reason why non-invasive bacteria secrete toxins that would interfere with this process remains puzzling. This study on cell responses to Bin, and previous work with VCC or VacA, establish that autophagy forms part of the arsenal of responses to bacterial pore-forming intoxication. Future studies will help to identify the signal(s) and the cell sensor(s) that elicit(s) this pathway.

## Materials and Methods

### Cell culture, transfection, and treatment

Strain II MDCK cells expressing the full-length CPM1 cDNA sequence (MDCK-Cpm1 cells) were generated as previously described [12], and cultured in Eagle's minimum essential medium (MEM) supplemented with 5% fetal calf serum and 400 µg ml^−1^ G-418 at 37°C in a humidified atmosphere containing 5% CO_2_. Transfections were performed using Lipofectamine 2000 (Invitrogen) as a carrier according to the manufacturer's instructions. All the assays were performed 24 hours after transfection. To induce autophagy by amino acid starvation, cells were washed three times with Earle's balanced salts solution (EBSS, Gibco) and incubated in the same medium.

### Toxins

BinA- and BinB-glutathione-S-transferase fusion proteins were expressed in *E. coli* as reported earlier [49]. The activated forms were purified by affinity chromatography on Äkta-FPLC using GSTrap and Benzamidine-Sepharose columns (GE Healthcare, France). Purified BinA and BinB were stained with Alexa 488 and Alexa 543, respectively (Molecular Probes, France). All the intoxications were performed with an equimolar mixture (50 nM final concentration) of BinA and BinB subunits present in the medium for the whole experiment. This concentration was suitable to induce MDCK-Cpm1 cell vacuolisation.

### Plasmids, antibodies and fluorescent dyes

GFP-Rab 5 and GFP-Rab 7 fusion proteins, TfR-myc (a fusion of the transferrin receptor and the myc epitope), and GFP-Lamp1 (a fusion at the C-terminus of Lamp1 with GFP), were kindly provided by the INSERM U452, Nice, France. Rab4 fused with GFP was a gift from the INSERM U568, Nice, France. The constructs pEYFP-Golgi, pDsRed2-ER, and pDsRed2-Mitochondria were purchased from Clontech, France. GFP-LC3 (a fusion of the rat LC3 with GFP) was kindly provided by N. Mizushima (Tokyo Metropolitan Institute of Medical Science, Tokyo, Japan). The decay accelerating factor (DAF) fused to GFP (GPI-GFP) was from A. Galmiche (Max Planck Institut für Infektionsbiologie, Berlin, Germany). Phalloidin-TRITC and the mAb anti-β-tubulin were purchased from Sigma-Aldrich, France. The mAb anti-myc (clone 9E10) was purchased from Babco (Richmond, CA). The polyclonal rat anti-Cpm1 antibody was generated as previously described [5]. The Alexa 488-labeled anti-mouse secondary antibody and FITC-labeled anti-rat secondary antibody were purchased from Molecular Probes. Acidic compartments were labeled by incubating the cells with 1 µM LysoTracker (Molecular Probes) in the culture medium for 5 min at 37°C. After incubation, cells were washed with PBS and immediately processed for immunofluorescence analysis. DNA was stained with the monomeric cyanine nucleic acid stains TO-PRO-3 (Invitrogen).

### Immunofluorescence studies, microscopy techniques, and video imaging

Immunofluorescence studies were performed as previously described [12], [50]. After treatment MDCK cells were washed twice with PBS and fixed in PBS containing 2% (w/v) paraformaldehyde. For the immunofluorescence studies, cells were treated with PBS/0,1% TritonX-100, then blocked in PBS containing 2% (w/v) fish skin gelatine. Confocal images were obtained with an Axiovert 200 M microscope equipped with a confocal laser module LSM 510 META, using a 40× oil magnification lens (Carl Zeiss MicroImaging, Inc.). The images were combined and merged using Photoshop (Adobe software). All the confocal pictures represent single focal sections. Videomicroscopy analyses were performed on an Axiovert 200 microscope equipped with shutter-controlled illumination (Carl Zeiss MicroImaging, Inc.) and a cooled digital charge-coupled device camera (Ropper Scientific). Images were processed using Metamorph 2.0 image analysis software (Invitrogen). For electron microscopy analysis, cells were fixed with 1.6% glutaraldehyde in 0.1M Phosphate buffer pH 7.4 at 22–24°C, then at 4°C for at least 18 hours. Samples were rinsed in the same buffer, then post-fixed with 1% osmium tetroxide and 1% potassium ferrocyanide for 1h at 22–24°C to enhance the staining of cytoplasmic membranes (de Bruijn, 1973). Cells were rinsed with distilled water and embedded in epoxy resin. Embedded samples were then conventionally processed for transmission electron microscopy (CM12; Philips).

### Statistical analysis

The effects of Bin intoxication or autophagy induced by starvation on both the number of autophagosomes per cell (Fig. 4A) and the percentage of GFP-LC3 positive cells (Fig. 4B) were evaluated with a one-way analysis of variance, and the Student's t-test with Bonferroni corrections was used for multiple comparisons between treatments. In order to evaluate the effect of the culture medium and the time of intoxication on the percentage of vacuolated cells (Fig. 4C), we used the Student's t-test. All analyses were performed on transformed data: square root transformation was used for the number of autophagosomes per cell and arcsine of square root transformation was used for percentages.

## Supporting Information

Figure S1 Figure S1 shows (A) microtubule network and (B) actin cytoskeleton analysis in Bin-treated cells. Cells were intoxicated with Bin for 6 h and processed for immunofluorescence analysis. Microtubules were visualized using anti-beta-tubulin mAb detected with a secondary antibody labeled with Alexa 488, and the actin cytoskeleton was visualized using TRITC-conjugated phalloidin. On middle confocal sections vacuoles (arrows) were found surrounded by actin. Bars, 10 μm.
(0.47 MB TIF)

Figure S2 MDCK-Cpm1 cells were transfected with markers of early and late endocytotic compartments. Twenty-four hours after transfection, cells were intoxicated with an equimolar mixture of unlabelled BinA and BinB-Al543 (50 nM) for 10 min at 37°C. GFP-Rab5 (A) and Cav1-GFP (B) served as markers for early endocytotic vesicles, GFP-Rab4 (C) is a typical marker of recycling compartments while TfR-myc (D) marks both endocytotic and recycling vesicles. At early time point of endocytosis we did not find any colocalization with the toxin and these markers.
(0.32 MB TIF)

Figure S3 Figure S3 shows MDCK-Cpm1 cells transfected with GFP-Rab7, GFP-Lamp1, GFP-Rab5 and GFP-Rab4. Twenty-four hours after transfection, cells were intoxicated with Bin specify which Bin eg BinA, BinB, BinA-Al498, BinB-Al543 and vacuolating cells were observed using a confocal microscope. The single focal sections and the total cell reconstitution show the association of GFP-Rab7 and GFP-Lamp1 with the membrane of the vacuolating compartment. In contrast, GFP-Rab5 and GFP-Rab4 were excluded from the vacuoles.
(0.08 MB JPG)

Figure S4 The upper panel shows MDCK-Cpm1 cells intoxicated with an equimolar mixture of unlabelled BinA- Al488 and BinB-Al543 (50 nM). After 6h of intoxication, the cells were processed for confocal microscopy. The single focal section and the total cell reconstitution show the colocalisation of the two subunits. The lower panel shows MDCK-Cpm1 cells transfected with the marker of late endocytotic compartments, GFP-Rab7. Twenty-four hours after transfection, cells were intoxicated with an equimolar mixture of unlabelled BinA and BinB-Al543 (50 nM). After 6h of intoxication, the cells were processed for confocal microscopy. The single focal section and the total cell reconstitution show i) that the membrane of the vacuoles are decorated by GFP-Rab7 ii) that the toxin is clustered in the remaining space between the vacuoles but is not associated with the membrane of these vacuoles and is not found inside the vacuoles.
(0.06 MB JPG)

Video S1 MDCK-Cpm1 cells treated with an equimolar mixture of BinA and BinB (50 nM) in complete medium at 37°C; the video starts 45 min after intoxication and ends 24 h after intoxication.
(2.50 MB MOV)

Video S2 Selection of video 1 showing two cells that progress through division and present vacuolation just after mitosis; the selection starts 15h after intoxication and ends 19 h after intoxication.
(1.19 MB MOV)

Video S3 The video shows two MDCK-Cpm1 cells that present vacuolation after mitosis when intoxicated in nutrient-free medium at 37°C; the video starts 20 h after intoxication.
(0.78 MB MOV)

Video S4 The video shows MDCK-Cpm1 cells treated with an equimolar concentration of BinA-Al488 and BinB-Al543 (50 nM) at 37°C that present post-mitotic vacuolation. For all videos, frames were collected every 15 min and are displayed at 3 frames/sec.
(0.16 MB MOV)

Video S5 Animations of the total cell reconstitution of GFP-Rab7 expressing MDCK-Cpm1 cells shown in figure S3.
(1.23 MB MOV)

Video S6 Total cell reconstitution of GFP-Rab5 exclusion of Bin induced giant vacuoles shown in fig. S3.
(0.16 MB MOV)

Video S7 Video S7 is an animation of figure S4 upper panel that shows a total cell reconstitution of BinAAl488 and BinBAl543 colocalisation in vacuolating cells.
(0.74 MB MOV)

Video S8 Video S8 is an animation of figure S4 lower panel that is a total cell reconstitution of BinBAl543 exclusion from GFP-Rab7 positive Bin induced giant vacuoles.
(1.08 MB MOV)
