# Supplementary figures and images for: Bacillus sphaericus Binary Toxin Elicits Host Cell Autophagy as a Response to Intoxication
Source: PLoS One. 2011 Feb 14;6(2):e14682. doi: 10.1371/journal.pone.0014682 (PMC3038859; doi:10.1371/journal.pone.0014682)

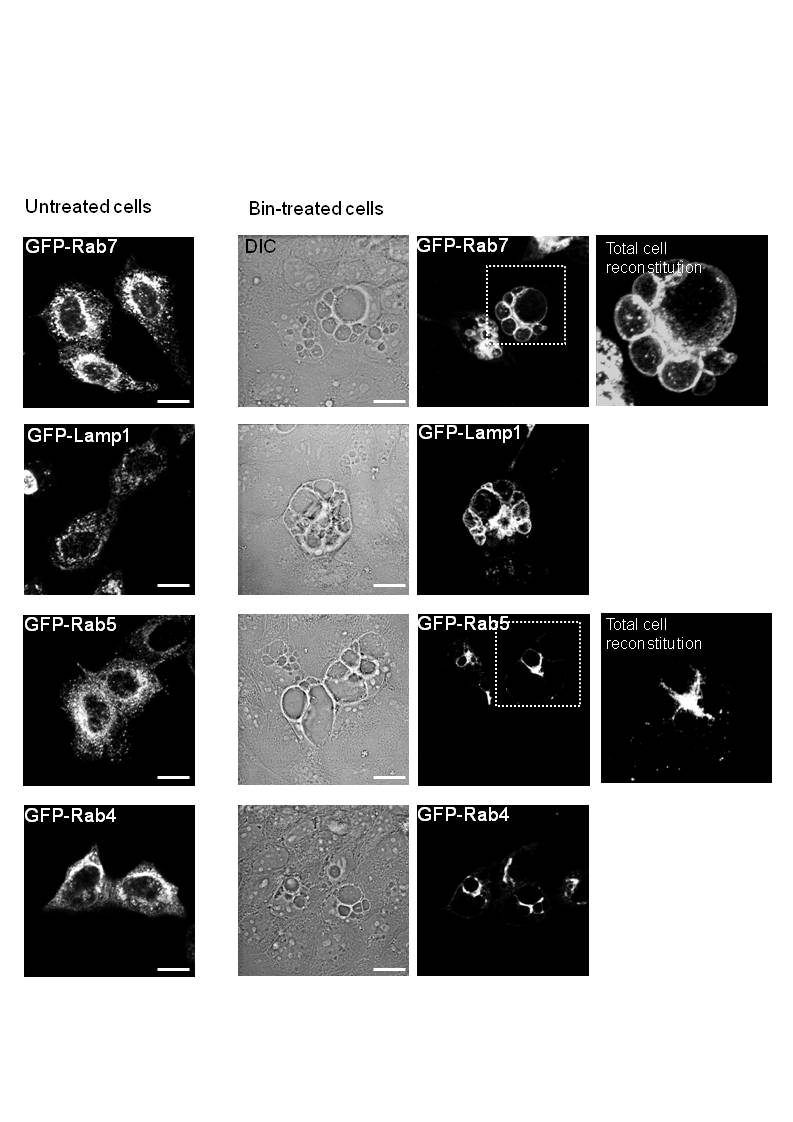

Supplement: Figure S3 — shows MDCK-Cpm1 cells transfected with GFP-Rab7, GFP-Lamp1, GFP-Rab5 and GFP-Rab4. Twenty-four hours after transfection, cells were intoxicated with Bin specify which Bin eg BinA, BinB, BinA-Al498, BinB-Al543 and vacuolating cells were observed using a confocal microscope. The single focal sections and the total cell reconstitution show the association of GFP-Rab7 and GFP-Lamp1 with the membrane of the vacuolating compartment. In contrast, GFP-Rab5 and GFP-Rab4 were excluded from the vacuoles. (0.08 MB JPG) [file pone.0014682.s003.jpg]

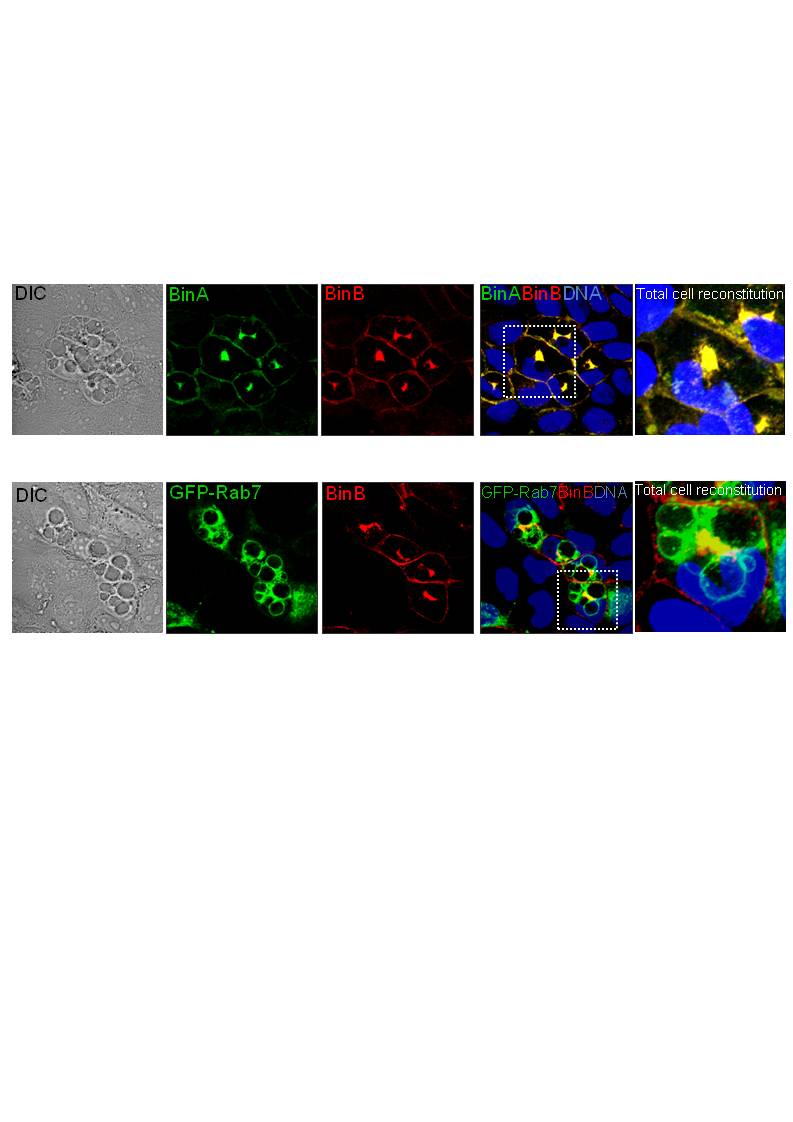

Supplement: Figure S4 — The upper panel shows MDCK-Cpm1 cells intoxicated with an equimolar mixture of unlabelled BinA- Al488 and BinB-Al543 (50 nM). After 6h of intoxication, the cells were processed for confocal microscopy. The single focal section and the total cell reconstitution show the colocalisation of the two subunits. The lower panel shows MDCK-Cpm1 cells transfected with the marker of late endocytotic compartments, GFP-Rab7. Twenty-four hours after transfection, cells were intoxicated with an equimolar mixture of unlabelled BinA and BinB-Al543 (50 nM). After 6h of intoxication, the cells were processed for confocal microscopy. The single focal section and the total cell reconstitution show i) that the membrane of the vacuoles are decorated by GFP-Rab7 ii) that the toxin is clustered in the remaining space between the vacuoles but is not associated with the membrane of these vacuoles and is not found inside the vacuoles. (0.06 MB JPG) [file pone.0014682.s004.jpg]
